# Prediction of Suitable Habitat Distribution of *Cryptosphaeria pullmanensis* in the World and China under Climate Change

**DOI:** 10.3390/jof9070739

**Published:** 2023-07-11

**Authors:** Chengcai Yan, Haiting Hao, Zhe Wang, Shuaishuai Sha, Yiwen Zhang, Qingpeng Wang, Zhensheng Kang, Lili Huang, Lan Wang, Hongzu Feng

**Affiliations:** 1 Key Laboratory of Integrated Pest Management (IPM) of Xinjiang Production and Construction Corps in Southern Xinjiang, College of Agronomy, Tarim University, Alar 843300, China; yancc119@126.com (C.Y.); m18919046163@163.com (H.H.); kangzs@nwsuaf.edu.cn (Z.K.); huanglili@nwafu.edu.cn (L.H.); 2 Scientific Observing and Experimental Station of Crop Pests in Alar, Ministry of Agriculture, College of Agronomy, Tarim University, Alar 843300, China; 3 The National and Local Joint Engineering Laboratory of High Efficiency and Superior-Quality Cultivation and Fruit Deep Processing Technology of Characteristic Fruit Trees in Southern Xinjiang, Alar 843300, China; 4 State Key Laboratory of Crop Stress Biology for Arid Areas, Northwest A&F University, Yangling 712100, China; 5 Yangling Seed Industry Innovation Center, Northwest A&F University, Yangling 712100, China

**Keywords:** *Cryptosphaeria pullmanensis*, MaxEnt model, global climate change, habitat shift, population distribution

## Abstract

Years of outbreaks of woody canker (*Cryptosphaeria pullmanensis*) in the United States, Iran, and China have resulted in massive economic losses to biological forests and fruit trees. However, only limited information is available on their distribution, and their habitat requirements have not been well evaluated due to a lack of research. In recent years, scientists have utilized the MaxEnt model to estimate the effect of global temperature and specific environmental conditions on species distribution. Using occurrence and high resolution ecological data, we predicted the spatiotemporal distribution of *C. pullmanensis* under twelve climate change scenarios by applying the MaxEnt model. We identified climatic factors, geography, soil, and land cover that shape their distribution range and determined shifts in their habitat range. Then, we measured the suitable habitat area, the ratio of change in the area of suitable habitat, the expansion and shrinkage of maps under climate change, the direction and distance of range changes from the present to the end of the twenty-first century, and the effect of environmental variables. *C. pullmanensis* is mostly widespread in high-suitability regions in northwestern China, the majority of Iran, Afghanistan, and Turkey, northern Chile, southwestern Argentina, and the west coast of California in the United States. Under future climatic conditions, climate changes of varied intensities favored the expansion of suitable habitats for *C. pullmanensis* in China. However, appropriate land areas are diminishing globally. The trend in migration is toward latitudes and elevations that are higher. The estimated area of possible suitability shifted eastward in China. The results of the present study are valuable not only for countries such as Morocco, Spain, Chile, Turkey, Kazakhstan, etc., where the infection has not yet fully spread or been established, but also for nations where the species has been discovered. Authorities should take steps to reduce greenhouse gas emissions in order to restrict the spread of *C. pullmanensis*. Countries with highly appropriate locations should increase their surveillance, risk assessment, and response capabilities.

## 1. Introduction

Canker and dieback are considered to be devastating fungal diseases of woody plants that can result in significant economic and ecological losses for orchards and forest ecosystems [1]. Airborne fungal pathogen *Cryptosphaeria pullmanensis* causes canker disease in crops and forest plant, which was first recorded and described on fallen *Populus trichocarpa* branches in the United States in 1984 [2], and was subsequently found in California, Nevada, Washington, etc. [3,4]. This fungus primarily causes canker on the branches of *P. fremontii* [3], *Vitis vinifera*, *P. deltoides* [4], *P. nigra* [5,6], *Salix alba*, *P. alba* [6,7], *P. euphratica* [6,8], *Elaeagnus angustifolia* [9], *Tilia cordata* Mill [9], and *Juglans regia* [10,11]. The species has effectively established itself in China [7,11,12,13] and Iran [5,10] over the past decade, where it was not previously found. According to the literature reports, *C. pullmanensis* was first discovered in 2016 on *Populus alba*, *P. nigra*, *S. alba,* and *S. matsudana* in Xinjiang Uygur Autonomous Region, China [6,7]. Since then, *C. pullmanensis* was successively discovered on *T. cordata* Mill [9], *P. euphratica* in [6,9], *E. angustifolia* [9], and *J. regia* [11,14] in Xinjiang Uygur Autonomous Region and *P. alba* in Inner Mongolia Uygur Autonomous Region [12,13]. It suggests that *C. pullmanensis* may spread rapidly and the danger level is increasing in China. But to date, it is not clear when or how *C. pullmanensis* invaded China and how widespread the disease is in the country. As we all known, the Xinjiang Uygur Autonomous Region and Inner Mongolia Autonomous Region both comprise a fourth of China’s land area. Despite its vast distribution area, little is known about the factors that influence the existing and future distribution patterns of *C. pullmanensis* for the sake of its management and monitoring.

*Cryptosphaeria* canker is typically observed on woody plants damaged by biotic and abiotic stressors [15,16]; *C. pullmanensis* is no exception. *C. pullmanensis* can harm the bark, cambium, heartwood, sapwood, and other types of wood [3], typically killing young trees two to three years after infection, and older trees through disease, other infections, or abiotic stressors [7]. The plant pathogen’s spores may be dispersed by rain and wind and transported to new infection sites by insects, birds, and wind. Natural wounds (frost, hail, wind, and rain) and other methods of wound creation (insects and birds) make infection feasible [3]. *C. pullmanensis* has a severe impact in the western United States on native Fremont cottonwood trees, leading to the widespread decrease in Fremont cottonwood in California [3] and posing a significant danger to forestry production. In Iran, *C. pullmanensis* has also been identified in large numbers in *J. regia* [17] and *P. nigra* [10]. In this study, we pre-investigated 25 walnut orchards and its protective forest belt in the southern and eastern portions of Xinjiang Uygur Autonomous Region (Aksu Region, Kashgar Region, Hotan Region, Hami City, Bayingolin Mongolian Autonomous Prefecture) from 2019 to 2022 and found that *C. pullmanensis* is relatively common, particularly on *J. regia*, *P. euphratica*, *P. alba*, and *S. matsudana*, which was consistent with previous research.

Presently, *C. pullmanensis* research focuses mostly on host range, the detection of fungal pathogens [5,10,12], biological features [7,18], and whole genome analysis [11]. There is a lack of research on the epidemic of *C. pullmanensis.* The emergence of a plant disease epidemic is dependent on the presence of an aggressive pathogen, a susceptible host plant, and favorable environmental conditions [19]. Thus, It is critical to identify the primary environmental elements that contribute to plant disease prevalence. Climate, geography, soil, and land cover are the key factors restricting species dispersal [20]. Determining the probable geographic distribution of *Cryptosphaeria* canker is crucial for advising field management and monitoring strategies. To our knowledge, very few studies have attempted to forecast the possible dissemination of *C. pullmanensis*, especially potential developments in response to climate change. Instead, most surveys and studies of the disease have focused on orchards in small towns and rural villages.

It is well accepted that global temperature change would have a considerable effect on species distribution [21,22] and may exacerbate fungal diseases [20]. Due to the unique life cycles and growth patterns of fungi, however, few research works [23,24,25] have examined the distribution of fungi at vast spatial and temporal scales. The spatial data management and geographic information system (GIS) offer a potential answer to this issue [26,27,28]. Currently, a variety of species distribution models (SDMs) have been used to predict distribution area, ecological requirements and ecological response, including GARP (genetic algorithm for rule-set production) [29], CLIMEX (climate change experiment) [30], BIOCLIM (bioclimatic modeling) [31], GMPGIS (global geographic information system for a medicinal plant) [32], and MaxEnt (maximum entropy) [33,34]. Among these modeling approaches, the MaxEnt is an extensively used tool with superior predictive performance [35,36,37], and it has been demonstrated that it remains useful even when the distribution point’s number is imprecise and the correlation between climatic and environmental parameters is unpredictable [38,39]. For example, Zhang et al. [40] recently integrated GIS and MaxEnt methods to forecast the possibly appropriate locations for *Monilinia fructicola* under different climate change scenarios in China. Ruheili et al. also investigated the proportions and hotspots of witches’ broom disease using the future climate projections in Oman [41]. Therefore, immediate study is required to prevent the pandemic spread of *C. pullmanensis*, and it is critical to determine the possible danger locations and prevalence levels [42].

In our study, we combine a total of 76 distribution points (25 collected by ourselves; 51 obtained through the literature) of the *C. pullmanensis* combined with 34 environmental variables to forecast the probable global distribution variations of *C. pullmanensis* under various climatic circumstances using the MaxEnt model. In addition, we evaluated the direction of *C. pullmanensis* range alterations in response to climate change. The following are the objectives of the current study: (1) under climate change conditions, to simulate the probable geographic dispersion range of *C. pullmanensis* throughout the world and China; (2) to explore the primary climatic conditions that limit the potential distribution of *C. pullmanensis*; and (3) to develop a theoretical foundation for future *C. pullmanensis* prevention and management.

## 2. Materials and Methods

### 2.1. The Distribution Points of C. pullmanensis 

We used “*Cryptosphaeria pullmanensis*” as keywords to search the databases of the GBIF (Global Biodiversity Information Facility) (https://www.gbif.org/occurrence/search?taxon_key=5486752, accessed on 15 November 2022), Google Scholar (https://scholar.google.com.hk, accessed on 15 November 2022), and the CNKI (China National Knowledge Infrastructure) (https://www.cnki.net/, accessed on 16 November 2022), ultimately obtaining the complete literature occurrence points for the *C. pullmanensis*. Additionally, from 2019 to 2022, we conducted a field survey in China’s Xinjiang Uygur Autonomous Region and collected 25 occurrence sites using GPS. Subsequently, a thorough database of *C. pullmanensis* occurrences was created (Supplementary Table S1). The geographic distribution data for *C. pullmanensis* was then processed as follows. 

**Figure 1:**
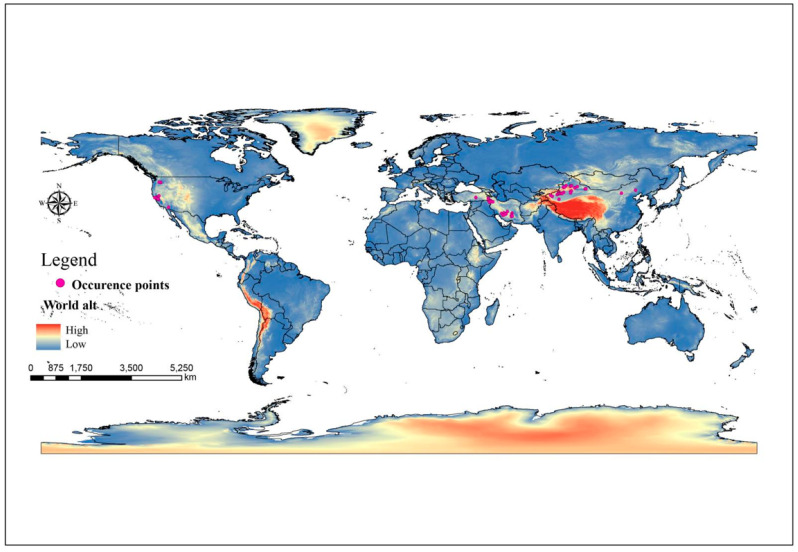
*C. pullmanensis* occurrence sites throughout the world were kept after spatial thinning with ENMTools with a 5 km buffer.

First, we used the online longitude and latitude query tool (www.mapcarta.com, accessed on 20 November 2022) to retrieve the exact longitude and latitude for a certain collection location (for example, hamlet or specific location) in the literature. Second, we converted the literature’s six-decimal latitudes and longitudes to a floating-point number (decimal system). Third, we ensured that the locations acquired individually matched the latitudes and longitudes reported in the literature. To reduce data sampling bias, we used the ENMTools.pl software (https://github.com/danlwarren/ENMTools, accessed on 26 November 2022) to trim occurrence points so that only one observation is maintained in each 2.5 arc min grid cell (approximately 5 km^2^) (matching to environment variable data below) [43]. Finally, *C. pullmanensis* had remaining occurrence points of 60 (Figure 1), providing enough data points to develop species distribution models [44]. The base map was from the standard map service system of the Ministry of Natural Resources as analysis base maps.

### 2.2. Environmental Factor Variables

The distribution of species is generally greatly influenced by climate conditions, habitat characteristics, terrain, and land cover [23,45], and various species have distinct environmental needs. We used soil conditions and vegetation conditions in this study as limiting ecological parameters since *C. pullmanensis* primarily damages woody plants. We first download 34 environmental variables (Supplementary Table S2) from the World Climate website (http://www.worldclim.org, accessed on 3 December 2022) with a spatial resolution of 2.5 min (approximately 5 km), which includes bioclimatic variables (Bio1-Bio19) and elevation. These variables may have an impact on the distribution of *C. pullmanensis*. They were analyzed using three separate socioeconomic models driven by CO_2_: shared socioeconomic pathways (SSPs) 126, 370, and 585 [46]. The BCC-CSM2-MR was utilized to perform this. ArcGIS 10.4.1 was used to determine the topographic information in the grid, which includes aspect and slope (ESRI, Redlands, CA, USA). Additionally, 11 soil factors were collected as the crucial environmental data using the Harmonized World Soil Database (http://www.fao.org/soils-portal/, accessed on 3 December 2022) for our study. Additionally, we obtained the worldwide land cover data for this study (around 1 km) from https://globalmaps.github.io (accessed on 5 December 2022). Using ArcGIS 10.4.1, all these data were transformed into ASCII format and their spatial resolution was unified into 2.5 arcminutes. We employed the same topography factors, land cover, soil data, and similar bioclimatic variables from different periods to forecast the future distribution of *C. pullmanensis* while taking into account the stability of the terrain, soil type, and land cover type [47].

We performed the jackknife analysis to determine the percentage contribution of each environmental variable in the MaxEnt program (version 3.4.4) because too many environmental variables can affect the ecological space dimension, in turn leading the accuracy of the results [45,48]. All 34 environmental variables were used for analysis using the ENMTools.pl software, and variables (correlation coefficient of more than 0.8) were eliminated [47]. Specifically, the variable was eliminated with a smaller contribution if the absolute value was >0.8 of the correlation coefficient between two variables. Both variables were kept if the correlation coefficient’s absolute value was less than 0.8 (Supplementary Figure S1) [49]. Therefore, for the purpose of building the species distribution model, we finally decided on 11 environmental factors (Supplementary Table S3).

### 2.3. Construction and Accuracy Evaluation of the MaxEnt Model

#### 2.3.1. Parameter Optimization of Maximum Entropy Model 

An appropriate parameter optimization approach was helpful in reducing mistakes due to model overfitting [50]. Aside from species occurrence data and environmental variables, the RM (regularization multiplier) (values ranging from 0.1 to 4, increments of 0.1) and FC (feature combination) (L: linear; Q: quadratic; H: hinge; P: product; T: threshold) [34] in the MaxEnt software are required to build the species distribution model. To find the best model tuning parameters for *C. pullmanensis*, we used the R software (version 3.6.3) with kuenm package (https://github.com/marlonecobos/kuenm, accessed on 12 November 2022) [51]. In general, the AIC (Akaike information criterion correction) is regarded as a criterion for evaluating the goodness of fitting statistical model since it takes into account the complexity of the model as well as the goodness of the model fitting data. This prioritizes the source with the lowest delta AICc model [52]. As a result, the model parameters were optimal when the rate of the omission was <5%, and the delta AICc was the minimum (<2 or =0) [51]. 

The filtered environmental variables and species distribution data were then put into the programs “Samples” and “Environmental layers,” and four future climatic scenarios (2030s, 2050s, 2070s, and 2090s) were forecasted. By choosing “Create response curves,” climate variables’ response curves were constructed, predictions were drawn, and Jackknife test results were used to assess the relative importance of environmental components [53]. Both the output format and the file type are set to “Logistic” and “asc”. To confirm the accuracy of the results, 10,000 background points and 10 replications (file type is subsample) were conducted. We set the RM = (1.1) and FC = (LQPT) parameters in MaxEnt models for *C. pullmanensis* based on the findings of model optimization (Supplementary Table S4). A total of 25% was chosen as the “Random test percentage” for the test data [53,54]. The “random seed” parameter has a random ratio set. The default values for the software’s other settings were used.

One of the best and most extensively used methods for evaluating the accuracy of niche models is the ROC (receiver operating characteristic) curve created by MaxEnt [43,44] by reducing false negative and false positive distribution findings. True skill statistic (TSS) and Kappa statistic were used to evaluate the accuracy of MaxEnt’s prediction [55]. To assess prediction accuracy, the AUC (the area under the receiver operating characteristic curve) is typically utilized [45]. The AUC value range is 0 to 1 [56]: ≤0.5 is regarded as poor prediction, >0.5 and ≤0.7 is regarded as acceptable prediction, >0.7 and ≤0.9 is regarded as good prediction, and >0.9 and ≤1 is regarded as outstanding prediction [57,58]. TSS was evaluated as excellent, 1.0–0.85; very good, 0.7–0.85; good, 0.55–0.7; fair, 0.4–0.55; and fail, <0.4 [59,60]. Kappa statistic was evaluated as excellent, >0.8; useful, 0.4–0.8; and poor, <0.4 [61].

#### 2.3.2. Classification of Potentially Suitable Areas

When used in conjunction with the reclassification command, the output data (“asc” file) from the MaxEnt model replication might be converted from ASCII to raster format using the ArcGIS format conversion tool. To categorize the model outputs, we utilized the maximum test sensitivity plus specificity (MTSPS) [37,62] threshold and constructed binary maps (suitable or not suitable). The distribution map was then classed as no suitability *p* ≤ 0.1915 (MTSPS), low suitability 0.1915 < *p* ≤ 0.4, medium suitability 0.4 < *p* ≤ 0.6, and high suitability *p* > 0.6, respectively.

### 2.4. Statistical and Spatial Analysis

#### 2.4.1. Calculating Distribution Shifts

Using SDM toolbox (version 2.4), changes in possible distribution areas were computed after modeling the present and future appropriate suitable area for *C. pullmanensis* [63]. We compared future acceptable suitable areas to the present distribution to determine zones that were: (1) expanded, (2) stable, and (3) shrunken. The area of the regions selected in steps 1 through 3 was then computed.

#### 2.4.2. Centroid Migration 

To further investigate the dynamic shift pathways of *C. pullmanensis*, we initially calculated the centroid coordinates of *C. pullmanensis* in different time periods (present, 2030s, 2050s, 2070s, and 2090s) and SSP (SSP126, SSP370, and SSP585) using SDM toolbox (version 2.4) [63]. Second, The centroid’s migration trajectory was analyzed by creating a vector file that depicted the direction and magnitude of changes over time [64]. Thirdly, migration distance was determined by comparing the centroids of several time periods (current—2030s, current—2050s, current—2070s, and current—2090s) in various climate scenarios. All of our geographical maps were projected using the Asia North Albers equal area conic projection to accommodate the study area’s location.

## 3. Results

### 3.1. Assessment of the Model’s Accuracy

MaxEnt predicted the potentially suitable area for *C. pullmanensis* in China using 60 distribution records and 11 environmental variables, and the minimal AICc was used to choose the best feature combination. After optimization, the parameter RM was set to 1.1, the FC to LQPT, and the delta AICc to 0. (Supplementary Table S4). The MaxEnt model runs 10 times with optimal parameters (Supplementary Figure S2). The optimized MaxEnt model performed remarkably well in predicting the potentially suitable area for *C. pullmanensis*, as shown by the average training AUC (Supplementary Figure S3) of 0.9904 and the average test AUC of 0.978. Both values were greater than 0.9 and greater than the AUC value corresponding to random classification (0.5). The TSS and Kappa values were 0.938 and 0.937, respectively, demonstrating that the optimized MaxEnt model performed well in predicting the potentially suitable area for *C. pullmanensis*.

### 3.2. Environmental Variable Analysis for the Identification of the Predicted Potentially Appropriate Area of C. pullmanensis

Detailed jackknife testing and a percent contribution study revealed that annual mean temperature (bio1, 35.4%), the precipitation of the warmest quarter (bio18, 29.4%), the precipitation of the driest month (bio14, 15.3%), land cover (6%), and elevation (5.4%) had the greatest influence on *C. pullmanensis* distribution (Supplementary Table S5). The total percentage contribution was 91.5%, while the total permutation importance was 93.4%. Out of the eleven environmental variables, precipitation had the greatest impact, followed by temperature, land cover, and elevation; on the other hand, soil and terrain both had a relatively small impact on the distribution of *C. pullmanensis* distribution.

Notably, the study conducted an independent investigation of the influence of prospective habitats using environmental parameters with a contribution rate of more than 6% (Supplementary Figure S5). A higher or lower annual mean temperature range (<5.7 °C or >17.3 °C) will affect the adaptability of *C. pullmanensis*. The likelihood of encountering *C. pullmanensis* reduces as precipitation increases. The *C. pullmanensis* is particularly sensitive to precipitation, and a lack of precipitation aids in its spread. When the precipitation of the warmest quarter (bio 18) exceeds 71.3 mm and the precipitation of the driest month (bio 14) exceeds 6.7 mm, the environment is no longer adequate for its growth. *C. pullmanensis*, on the other hand, is relatively less affected by terrain or soil.

### 3.3. Current Potentially Suitable Habitats of C. pullmanensis in the World and China

#### 3.3.1. Global Suitable Habitats under Current Climate Scenario Models

**Figure 2:**
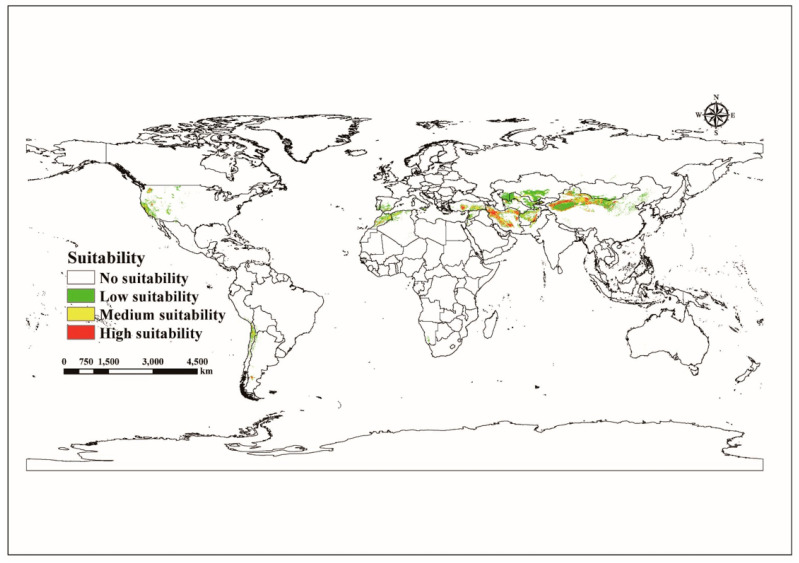
Global suitable habitats under current climate scenario models (no suitability *p* ≤ 0.1915; low suitability 0.1915 < *p* ≤ 0.4; medium suitability 0.4 < *p* ≤ 0.6; high suitability *p* > 0.6, *p* = probability).

The total area of all globally eligible regions, as shown by Figure 2 and Supplementary Table S6, is 5.38 × 10^6^ km^2^. The most favorable places are mostly in East Asia (China), West and Central Asia (Iran, Afghanistan, and Turkey), South America (Chile and Argentina), and California’s West Coast in the United States. The high-appropriate areas cover a total world area of 0.59 × 10^6^ km^2^. Southern Kazakhstan, most of Uzbekistan, northwestern (mainly in Tarim basin and Gansu Corridor) and northern (most of Inner Mongolia Autonomous Region) China, southern Spain, most of Morocco, northern Algeria, most of Greece, west and mid-west of the United States, most of Chile, southern Syria, and most of Afghanistan have low suitable areas. The global low suitable areas cover a total area of 3.37 × 10^6^ km^2^. The medium suitable areas are roughly spread between the high and low suitable zones. The global low suitable zones cover a total area of 1.42 × 10^6^ km^2^.

#### 3.3.2. Current Potentially Suitable Habitats of *C. pullmanensis* in China

**Figure 3:**
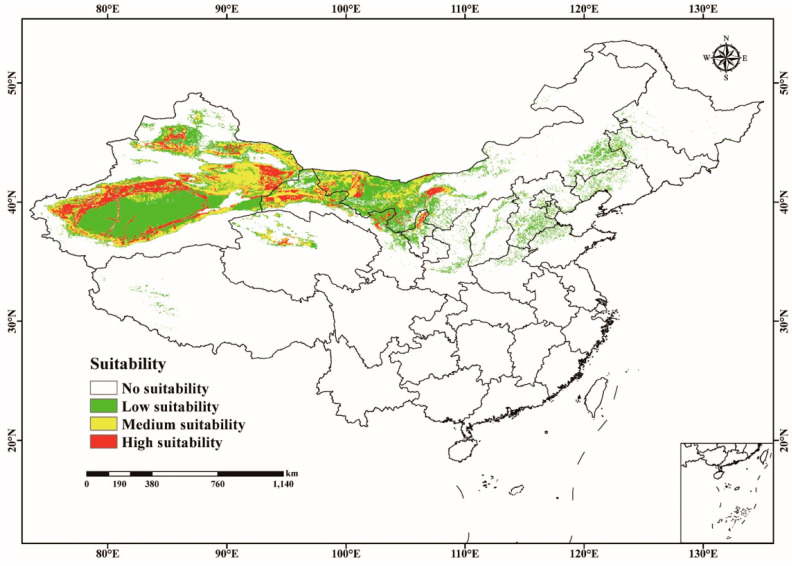
The current potentially geographical distribution of *C. pullmanensis* in China (no suitability *p* ≤ 0.1915; low suitability 0.1915 < *p* ≤ 0.4; medium suitability 0.4 < *p* ≤ 0.6; high suitability *p* > 0.6, *p* = probability).

Figure 3 and Supplementary Table S7 show that the predicted potentially suitable locations for *C. pullmanensis* in China are primarily distributed in 11 provinces, including Xinjiang Uygur Autonomous Region, Qinghai, Inner Mongolia Autonomous Region, Gansu, Ningxia, Shanxi, Shannxi, Hebei, Liaoning, Jilin, and Tibet. The majority of Ningxia and the western portion of Gansu are also found in these regions. Distributions are sporadic in northern Qinghai, southern Tibet, northern Shanxi, the bulk of Hebei, and the westernmost portions of Liaoning and Jilin, and are found mostly in the whole of Xinjiang Uygur Autonomous Region, west of Gansu (mainly in Gansu Corridor), and most of Inner Mongolia and Ningxia. Northern Qinghai, southern Tibet, northern Shannxi, the majority of Hebei, and west of Liaoning and Jilin all have sporadic distributions.

The majority of Xinjiang Uygur Autonomous Region, western Gansu, Midwest Inner Mongolia Autonomous Region, Northern Qinghai, and Ningxia had high suitability areas for *C. pullmanensis*, and the entire area was 24.96 × 10^4^ km^2^, accounting for 2.6% of China’s total territory. *C. pullmanensis* medium-suitability habitats were frequently next to high-suitability habitats, with a total area of 43.89 × 10^4^ km^2^, accounting for 4.57% of the research area. The remaining suitable areas are low-suitable habitats, with a total area of 99.07 × 10^4^ km^2^, accounting for 10.32% of the research area. Furthermore, other provinces and cities were unfavorable places for *C. pullmanensis*, with a total size of 792.08 × 10^4^ km^2^, accounting for 82.51% in China.

### 3.4. Future Potentially C. pullmanensis Habitats in the World and China

#### 3.4.1. Global Suitable Areas under Future Climate Scenario Models

**Figure 4:**
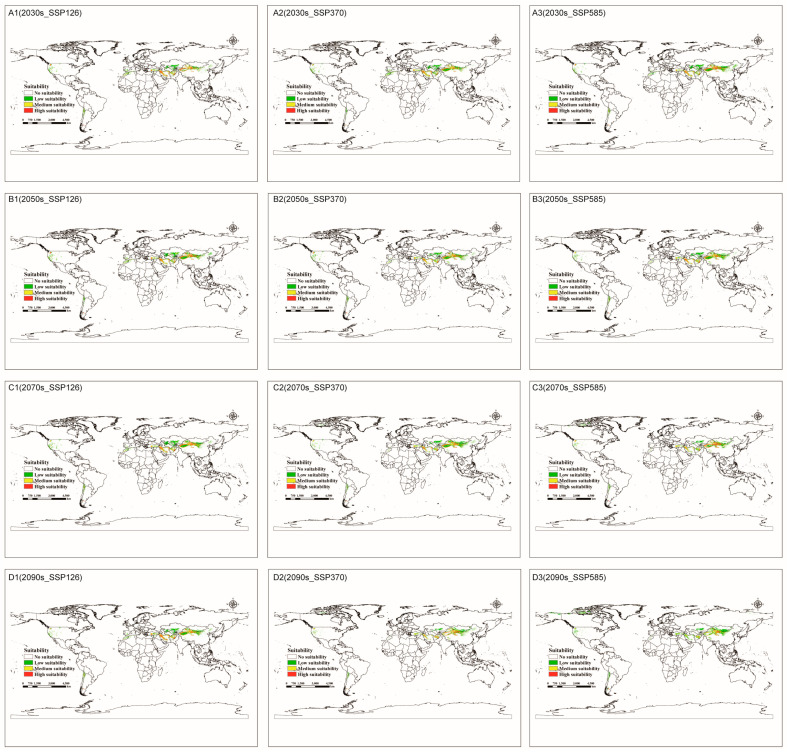
The future potentially geographical distributions of *C. pullmanensis* in the world under future climatic conditions. (**A1**–**A3**) Potential habitats of *C. pullmanensis* in the world in the 2030s under the SSP126, SSP370, and SSP585 scenarios. (**B1**–**B3**) Potential habitats of *C. pullmanensis* in the world in the 2050s under the SSP126, SSP370, and SSP585 scenarios. (**C1**–**C3**) Potential habitats of *C. pullmanensis* in the world in the 2070s under the SSP126, SSP370, and SSP585 scenarios. (**D1**–**D3**) potential habitats of *C. pullmanensis* in the world in the 2090s under the SSP126, SSP370, and SSP585 scenarios.

Figure 4 and Supplementary Table S6 show that the global trend for suitable land area is generally decreasing. In the SSP370 and SSP585 scenarios, the two-period total usable area declined significantly (maximum reduction of 55.58%) and showed a modest northward shift in the northern hemisphere. The clearest indication was the establishment of suitable regions in northern Canada (Figure 4(C2,C3,D2,D3)). In the SSP126 scenario, however, the total acceptable area decreased somewhat, and the overall changes are insignificant. All future changes in high-suitability areas show a slight increase trend only in the SSP126 scenario.

#### 3.4.2. Potentially Suitable Habitats for *C. pullmanensis* Based on Future Climatic Scenarios in China

**Figure 5:**
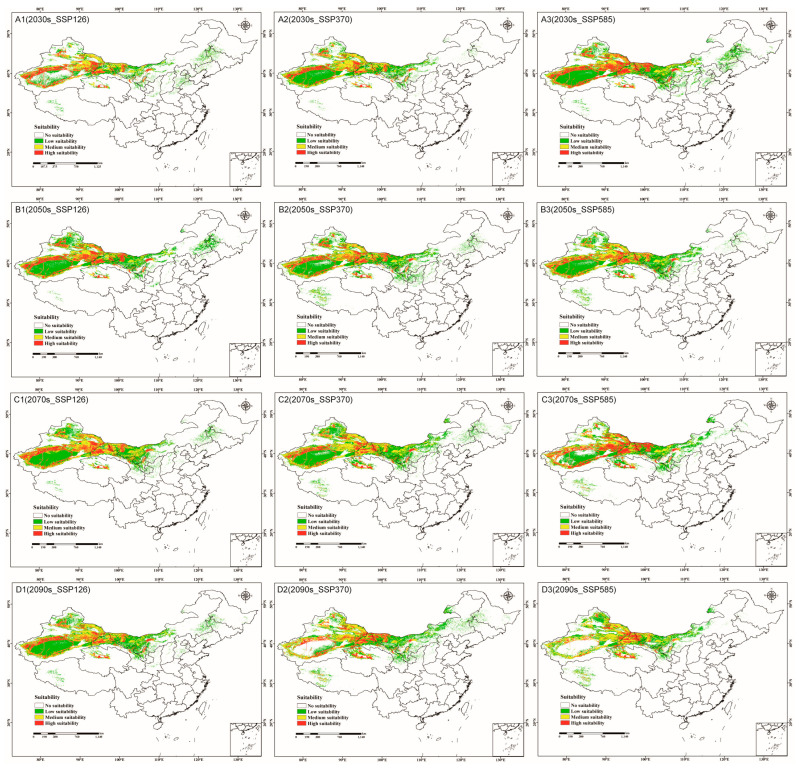
The future potentially geographical distributions of *C. pullmanensis* in China under future climatic conditions. (**A1**–**A3**) Potential habitats of *C. pullmanensis* in China in the 2030s under the SSP126, SSP370, and SSP585 scenarios. (**B1**–**B3**) Potential habitats of *C. pullmanensis* in China in the 2050s under the SSP126, SSP370, and SSP585 scenarios. (**C1**–**C3**) Potential habitats of *C. pullmanensis* in China in the 2070s under the SSP126, SSP370, and SSP585 scenarios. (**D1**–**D3**) potential habitats of *C. pullmanensis* in China in the 2090s under the SSP126, SSP370, and SSP585 scenarios.

Figure 5 and Supplementary Table S7 illustrate the circumstances of suitable *C. pullmanensis* habitats during various future eras and climate scenarios. Middle and high-appropriate habitats for *C. pullmanensis* in China are primarily located along the edges of the Tarim basin and Junggar basin, eastern Xinjiang, Gansu Corridor, Midwest Inner Mongolia, and north of Qinghai and Ningxia under each future climatic scenario model. And the low-suitable regions are primarily located in the central Tarim basin and Junggar basin, southwestern Tibet, and eastern Inner Mongolia. The suitable area is increasing as a whole in China; among them include the fact that the future changes in total appropriate areas indicated a dropping and then rising tendency in the SSP126 and SSP370 conditions. However, in the SSP585 scenario, the overall area of eligible regions expanded at first and then reduced in comparison to the current condition. In addition, the maximum suitable area appeared in the 2070s (183.66 × 10^4^ km^2^) under the SSP126 scenario, the maximum suitable area appeared in the 2070s (204.25 × 10^4^ km^2^) under the SSP370 scenario, and the maximum suitable area appeared in the 2030s (202.67 × 10^4^ km^2^) under the SSP585 scenario.

**Figure 6:**
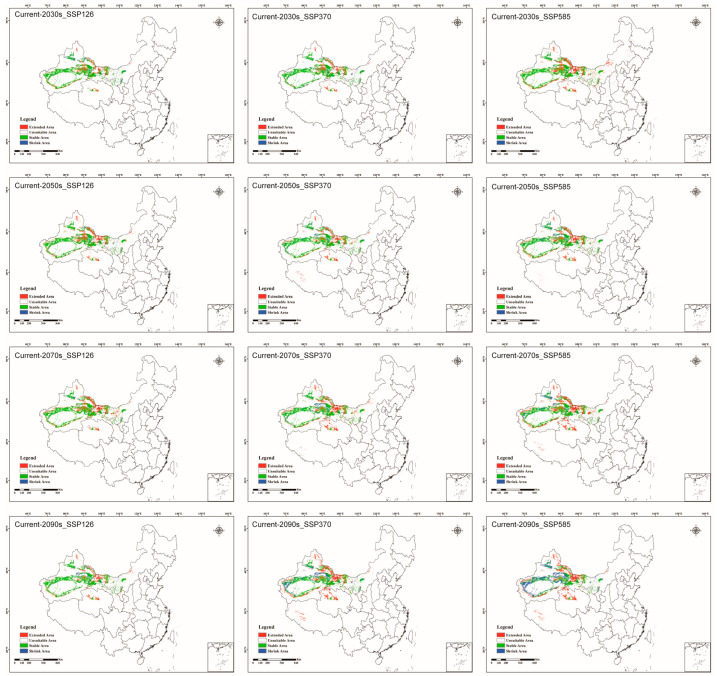
Adaptability changes in *C. pullmannensis* under different future climate scenarios (the scenario of the first row is SSP126, the scenario of the second row is SSP370, and the scenario of the third row is SSP585).

Figure 6 and Supplementary Table S8 illustrate the adaptability changes in *C. pullmannensis* under different future climate scenarios (Figure 3). *C. pullmanensis* has significantly expanded its range in the north of Junggar basin, the Tarim basin’s edge, eastern Xinjiang, northwestern Gansu, northern Qinghai, and southwestern Tibet. Under the SSP370 and SSP585 scenarios for the 2090s, the predominant shrinkage area is the margin of the Tarim basin, indicating that the environmental conditions in this region are not suitable for the life of *C. pullmanensis.*

### 3.5. Centroid Migration of Potential Suitable areas for C. pullmanensis Based on Future Climatic Scenarios 

**Figure 7:**
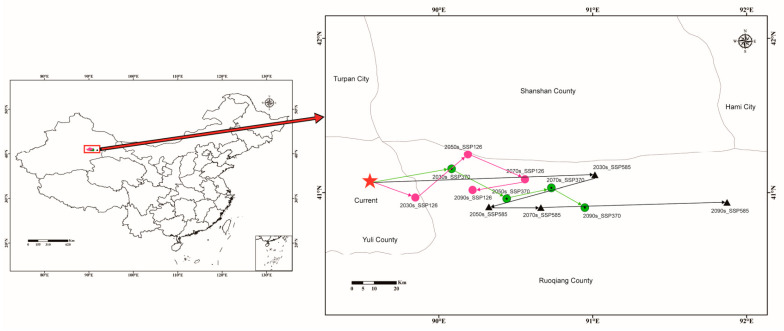
Centroid shifts of potential suitable area for *C. pullmanensis* under different climatic scenarios in China. Red star indicates the centroids of the suitable habitats of *C. pullmanensis* under current climate. Dots and triangles represent the centroids of the suitable habitats of *C. pullmanensis* under different future climate scenarios.

Due to the uneven form of the acceptable habitat areas for *C. pullmanensis*, we utilized a centroid migration analysis to evaluate the changes in the distribution pattern under twelve climate change scenarios. We found that the core of the *C. pullmanensis* acceptable habitat areas under the current climate change scenario is situated in Yuli county, in southern Xinjiang Uygur Autonomous Region (89.552582°, 41.070778°). Under various climate change scenarios, it was expected that the center of appropriate habitat areas will shift eastward. 2090s_SSP585 predicted the most distant migration (91.872354°, 40.936859°), followed by 2090s_SSP370 (90.948488°, 40.901504°). However, according to the SSP126 scenario, *C. pullmanensis* was projected to move eastward in the 2070s and subsequently westward in the 2090s (90.218878°, 41.016473°) (Table 1 and Figure 7). 

## 4. Discussion

### 4.1. Accuracy of MaxEnt after Optimization

MaxEnt can build species response curves, objectively analyze the environmental characteristics of pertinent habitats, and is unaffected by sample size [65]. Based on the results of this experiment, 60 occurrence points were adequate data to create species distribution models [44]. Typically, the species and its data structure define the model parameters [66]. The bulk of earlier studies used the MaxEnt model with default settings, which led to overfitting and sampling bias and had a detrimental effect on the capacity to generalize species predictions [67]. The kuenm package was used in this experiment to improve the MaxEnt model because it reduces the likelihood of overfitting the model by matching and analyzing species distribution data with environmental variables and maintaining an effective distribution data in a similar niche [51]. After changing the RM from 1 to 1.1 and the FC from LQHPT to LQPT, the AICc decreased from 3051.2 to 0, showing that there was less overfitting as a result of optimization. Following optimization, the AUC, TSS, and Kappa scores were 0.9904, 0.938, and 0.937, respectively, indicating excellent results accuracy.

### 4.2. The Effect of Environmental Factors on the Distribution of C. pullmanensis

Due to their distinct development forms, fungi cannot thrive in the absence of proper flora and soil conditions, even if the climate and terrain are ideal [20]. Therefore, an excessive amount of environmental variables and parameters need to be taken into account. Species distribution may be disproportionately impacted by environmental variables [68]. Our findings show that climate factors influence the spread of *C. pullmanensis* more strongly than topography, land cover, and soil factors, and the temperature variables (bio1) have a more significant effect than precipitation variables (bio14 and bio18). Although the total contribution rate is only 19.9%, the role of topography, land cover, and soil factors cannot be ignored for the distribution and growth of *C. pullmanensis*. We hypothesized two reasons, the first of which focuses on the influence scale of environmental variable type. Changes in soil factors, land cover, and topographic factors often affect the distribution of species on a narrow spatial scale, while climate factors are the opposite. Furthermore, in comparison to climate conditions, topography, land cover, and soil variables have an impact on the pathogen’s host. The second explanation is related to the infection approach. Previous investigations have proven that wounds under biotic (i.e., wounds produced by pruning, insects, birds, etc.) or abiotic (i.e., wounds produced by frost, hail, wind, rain, or the natural shedding of catkin, etc.) stress [15,16] are the main path of *C. pullmanensis* infection. Northwestern and northern China are dominated by temperate continental climate and temperate monsoon climate, with low annual rainfall, large radiation angles, seasonally high temperatures [69], cold and dry winters, sparse and solitary vegetation, a high degree of salinization and alkalinity [70,71], and the main hosts of *C. pullmanensis* such as *S. alba*, *P. alba*, *S. matsudana*, *J. regia*, *P. euphratica*, and *E. angustifolia* are widely distributed. Under such natural environmental conditions, the occurrence of forest wounds is inevitable. In addition, the optimal temperature for *C. pullmanensis* growth is 28–31 °C in darkness, but the fungus can still grow slowly at 37–40 °C or 0–15 °C [7]. Compared to acidic settings, *C. pullmanensis* favors alkaline habitats [7]. It indicates that *C. pullmanensis* can adapt to extreme temperatures and high salinity environments in northwestern and northern China. However, southern China is dominated by a subtropical monsoon climate, with a high annual precipitation and warm and humid winters, which indicates that it is unsuitable for *C. pullmanensis* or that suitability is limited in southern China.

### 4.3. Changes in the Distribution of C. pullmanensis in the Future 

According to the IPCC (2021) assessment, yearly precipitation would increase by 5 to 7% by 2050, and the average annual temperature may rise by 2.3 to 3.3 °C in China [72]. In other words, China’s climate may become warmer and wetter in the future. According to predictions, by the end of this century, global warming will be between 1.6 °C and 5 °C, and annual precipitation will increase by 1.5% to 20% [73]. According to our results, the predicted potential suitable areas for *C. pullmanensis* based on the present climate conditions were primarily located in America, Iran, and China, and the majority can be found in China’s Xinjiang Uygur Autonomous Region and Inner Mongolia Autonomous Region, which is consistent with the results of previous resource surveys. The expected potential suitable areas for *C. pullmanensis* will shift poleward in latitude over time, based on several climate scenarios (Figure 4). However, the overall trend for suitable areas in China is increasing, and habitat fragmentation is a key issue. The centroids migrated to eastern China based on predicted climate conditions, and the pattern of migration based on high altitude was strong and clear. The growth in the area of high adaptability in the Tibetan Plateau, including Qinghai province and the Tibet Autonomous Region, is especially notable (Figure 6). Along the Tianshan Mountains, Pamir Mountains, Kunlun Mountains, Altai Mountains, Altun Mountains, Qilian Mountains, and Liupan Mountains, appropriate habitats have also increased dramatically. In addition, based on the SSP370 scenario from 2070, the distribution pattern of *C. pullmanensis* has changed the most due to climate change, indicating that the predicted potential suitable area for *C. pullmanensis* may expand in the future due to increased temperature and precipitation.

### 4.4. Limitations of SDM in Predicting Species Distribution

Ecological niche models demonstrated good prediction power in identifying currently occupied and unoccupied areas for *C. pullmanensis*. Our models can be used to analyze species distribution as well as to design prevention efforts toward the most critical places in priority management areas and non-priority management areas. Nonetheless, the present results may have also been affected by a number of uncertainties. Firstly, the precision of species occurrence data, particularly from published sources, increases the predictability uncertainty. Because certain distribution locations lacked latitudes and longitudes, they were found by searching for place names using coordinate positioning software, which may have led to geographical mistakes. Secondly, the incidence and prevalence are not only influenced by climate-causing plant diseases, but also by host conditions and medication frequency. In addition to soil type, climate, vegetation type, and topographic parameters utilized in this study, variety type, human activities, species interactions, and socioeconomic structure can affect host distribution [74,75]. Therefore, the anticipated potential area of suitability will differ from the actual area of suitability. In addition, the present study focused solely on the spatial ecology and not the epidemiological elements; hence, the results do not indicate the likelihood of illness occurrences. 

In order to more precisely direct *C. pullmanensis* surveillance, early warning, and prevention in future studies, on the one hand, it is vital to acquire an extensive amount of field survey data, and on the other, refining the algorithms to employ more relevant information and improving the model’s intelligence would further improve the simulation’s accuracy.

## 5. Conclusions 

In this study, for the first time, the MaxEnt model with optimized parameters was used to simulate the distribution of possibly appropriate habitats for *C. pullmanensis* based on current climate conditions and projected climate change. Temperature was the most significant element (threshold) influencing its distribution, followed by precipitation and land cover, such as annual mean temperature (5.7–17.4 °C), the precipitation of the driest quarter (<71.3 mm), and the precipitation of the driest month (<6.7 mm). Combining the response curves of the main climate parameters of *C. pullmanensis*, warm and dry areas within a specific temperature range are more conducive to the growth of *C. pullmanensis*. For the current climate, *C. pullmanensis* is mostly found in northwestern China, the majority of Iran, Afghanistan, and Turkey, northern Chile, southwestern Argentina, and the west coast of California. As for China, the areas of low, medium, and high suitability for *C. pullmanensis* were 99.07, 26, and 24 square kilometers, respectively; the majority of the high-suitability areas were concentrated in the Xinjiang Uygur Autonomous Region and Inner Mongolia Autonomous Region in China. These regions have a total area of 167.92 × 10^4^ km^2^. Future temperature changes of various intensities will improve the *C. pullmanensis* suitable regions in China. However, the suitable areas of *C. pullmanensis* around the world are decreasing in number. The trend in migration is toward higher latitudes and altitudes. It is estimated that the center of the suitable area will move eastward in China. The results of this study will be valuable not only for nations such as Morocco, Spain, Chile, Turkey, Kazakhstan, etc., where the infection has not yet fully spread or been established, but also for countries where the species has been identified. Authorities should implement measures to minimize greenhouse gas emissions to prevent the spread of *C. pullmanensis*. Countries with highly appropriate locations should enhance surveillance, risk evaluation, and response.

## Figures and Tables

**Table 1:** Centroid shifts of potential suitable areas for *C. pullmanensis* under future climatic conditions.

Climate Scenario	Period	Centroid Coordinates		Direction	Migration Distance (between Two Adjacent Decades)/km
		Longitude /° E	Latitude /° N		
Current	1970–2000	89.552582	41.076586		
SSP126	2021–2040/2030s	89.847418	40.967573	Southeast	3.03
	2041–2060/2050s	90.188925	41.246166	Northeast	63.5
	2061–2080/2070s	90.559061	41.08451	Northeast	97.1
	2081–2100/2090s	90.218878	41.016473	Northeast	64.5
SSP370	2021–2040/2030s	90.082805	41.152546	Northeast	51.6
	2041–2060/2050s	90.440685	40.959847	Southeast	86.4
	2061–2080/2070s	90.73097	41.03039	Northeast	113.8
	2081–2100/2090s	90.948488	40.901504	Northeast	135.7
SSP585	2021–2040/2030s	91.013858	41.113851	Northeast	141
	2041–2060/2050s	90.324757	40.906896	Northeast	76.3
	2061–2080/2070s	90.662871	40.90278	Southeast	108.4
	2081–2100/2090s	91.872354	40.936859	Southeast	224.2

## Data Availability

Data will be made available on request.

## Supplementary Materials

The following supporting information can be downloaded at: https://www.mdpi.com/article/10.3390/jof9070739/s1, Figure S1: Correlation analysis of various environmental factors; Figure S2: Jackknife test of variable importance Regularized training gain; Figure S3: The receiver operating characteristic curve. Figure S4: Response curves for predictiors in MaxEne model; Table S1: Geographical distributions of C. pullmanensis species sampled in this study; Table S2: Environmental variables for current period model analysis; Table S3: The environmental variables used in this study; Table S4:The relevant parameters for the best model generated by kuenm; Table S5: Percent contribution and permutation importance of 11 variables; Table S6: The proportion of suitable areas for growing different grades of C. pullmanensis around world; Table S7: The proportion of suitable areas for growing different grades of C. pullmanensis in China; Table S8: Percentage of distrbution changes of C. pumanensis between current and future climate-change scenarios in China.
