# Supplementary material for: Prediction of Suitable Habitat Distribution of Cryptosphaeria pullmanensis in the World and China under Climate Change
Source: J Fungi (Basel). 2023 Jul 11;9(7):739. doi: 10.3390/jof9070739 (PMC10381404; doi:10.3390/jof9070739)
Supplement: Supplementary file 1 [file jof-09-00739-s001.zip › Table S1. Geographical distributions of C. pullmanensis species sampled in this study.pdf]

Table S1. Geographical distributions of *C. pullmanensis* species sampled in this study.

Species	Longitude (°E)	Latitude (°N)
Cryptosphaeria pullmanensis	118.91352	42.26268
Cryptosphaeria pullmanensis	111.75026	40.82625
Cryptosphaeria pullmanensis	86.18149	41.73237
Cryptosphaeria pullmanensis	80.06517	40.37579
Cryptosphaeria pullmanensis	86.788199	44.40984
Cryptosphaeria pullmanensis	86.21026	44.29055
Cryptosphaeria pullmanensis	80.84732	40.50143
Cryptosphaeria pullmanensis	86.02782	44.30300
Cryptosphaeria pullmanensis	81.67472	36.85722
Cryptosphaeria pullmanensis	75.99888	39.46638
Cryptosphaeria pullmanensis	83.94250	44.54611
Cryptosphaeria pullmanensis	81.52083	36.87444
Cryptosphaeria pullmanensis	82.49777	43.79083
Cryptosphaeria pullmanensis	83.14083	43.80611
Cryptosphaeria pullmanensis	80.26722	41.32055
Cryptosphaeria pullmanensis	80.23666	41.30166
Cryptosphaeria pullmanensis	80.45888	40.65166
Cryptosphaeria pullmanensis	82.68777	37.07805
Cryptosphaeria pullmanensis	76.91222	39.15361
Cryptosphaeria pullmanensis	80.35000	37.63833
Cryptosphaeria pullmanensis	82.788823	41.227749
Cryptosphaeria pullmanensis	82.955854	41.718278
Cryptosphaeria pullmanensis	79.40213	40.07555
Cryptosphaeria pullmanensis	81.30865	40.54907
Cryptosphaeria pullmanensis	80.59472	40.61166
Cryptosphaeria pullmanensis	80.45888	40.65166
Cryptosphaeria pullmanensis	81.56576	40.62759

Cryptosphaeria pullmanensis	81.85299	40.57433
Cryptosphaeria pullmanensis	81.36733	40.63154
Cryptosphaeria pullmanensis	86.54833	41.92944
Cryptosphaeria pullmanensis	86.953829	40.892112
Cryptosphaeria pullmanensis	86.11555	40.84527
Cryptosphaeria pullmanensis	93.8875	42.87944
Cryptosphaeria pullmanensis	52.8063	29.8763
Cryptosphaeria pullmanensis	52.151095	29.672723
Cryptosphaeria pullmanensis	53.6667	30.3333
Cryptosphaeria pullmanensis	52.6885	30.89258
Cryptosphaeria pullmanensis	-122.8473	38.5111
Cryptosphaeria pullmanensis	-121.9164	38.2219
Cryptosphaeria pullmanensis	-120.7679	37.1642
Cryptosphaeria pullmanensis	-116.0372	33.722
Cryptosphaeria pullmanensis	-121.9059	38.7185
Cryptosphaeria pullmanensis	-121.2774	37.937
Cryptosphaeria pullmanensis	-120.809	39.354
Cryptosphaeria pullmanensis	-122.8473	38.5111
Cryptosphaeria pullmanensis	-121.0501	37.5501
Cryptosphaeria pullmanensis	48.484467	36.674339
Cryptosphaeria pullmanensis	51.680374	32.661343
Cryptosphaeria pullmanensis	51.587524	30.668383
Cryptosphaeria pullmanensis	80.256854	41.164822
Cryptosphaeria pullmanensis	75.977464	39.480041
Cryptosphaeria pullmanensis	88.3048	43.363422
Cryptosphaeria pullmanensis	93.510432	42.885913
Cryptosphaeria pullmanensis	89.588055	44.036744
Cryptosphaeria pullmanensis	76.166152	39.735495
Cryptosphaeria pullmanensis	-120.63451	46.416520
Cryptosphaeria pullmanensis	-119.86781	46.13319

Cryptosphaeria pullmanensis	45.068333	37.549444
Cryptosphaeria pullmanensis	44.768611	38.196944
Cryptosphaeria pullmanensis	38.550556	38.550025
Cryptosphaeria pullmanensis	45.721944	36.765278
Cryptosphaeria pullmanensis	46.211111	36.521389
Cryptosphaeria pullmanensis	47.111667	36.400833
Cryptosphaeria pullmanensis	45.387778	36.955556
Cryptosphaeria pullmanensis	45.480000	36.155278
Cryptosphaeria pullmanensis	46.099444	36.969444
Cryptosphaeria pullmanensis	45.140300	36.6995
Cryptosphaeria pullmanensis	-117.1739	46.7304
Cryptosphaeria pullmanensis	51.581944	30.668056
Cryptosphaeria pullmanensis	54.444444	31.584167
Cryptosphaeria pullmanensis	56.414444	29.466667
Cryptosphaeria pullmanensis	56.476667	29.067778
Cryptosphaeria pullmanensis	56.571667	29.928333
Cryptosphaeria pullmanensis	56.914167	29.290833
Cryptosphaeria pullmanensis	56.813333	30.770556
