# Supplementary material for: Prediction of Suitable Habitat Distribution of Cryptosphaeria pullmanensis in the World and China under Climate Change
Source: J Fungi (Basel). 2023 Jul 11;9(7):739. doi: 10.3390/jof9070739 (PMC10381404; doi:10.3390/jof9070739)
Supplement: Supplementary file 1 [file jof-09-00739-s001.zip › Table S3 The environmental variables used in this study.pdf]

**Table S3. The environmental variables used in this study**

| Type | Code | Description | Unit |
|---|---|---|---|
| Climate | bio1 | Annual mean temperature | ℃ |
| | Bio14 | Precipitation of driest month | mm |
| | bio18 | Precipitation of the warmest quarter | mm |
| Soil | t-silt | Topsoil silt fraction | % |
| | t-teb | Topsoil TEB | cmol/kg |
| | t-ece | Topsoil electric conductivity | |
| | t-grave | Topsoil gravel content | % |
| Land cover | gm-lc-v3 | Land Cover Types (Cropland, Forest, Grassland, Shrubland, Wetland, Water, Tundra, Impervious Surface, Bare Land, Snow/Ice-Disturbed) | |
| Topographical | elev | Elevation | m |
| | slope | / | ° |
| | aspect | / | rad |
