# Supplementary material for: Prediction of Suitable Habitat Distribution of Cryptosphaeria pullmanensis in the World and China under Climate Change
Source: J Fungi (Basel). 2023 Jul 11;9(7):739. doi: 10.3390/jof9070739 (PMC10381404; doi:10.3390/jof9070739)
Supplement: Supplementary file 1 [file jof-09-00739-s001.zip › Table S4. The relevant parameters for the best model generated by kuenm.pdf]

Table S4. The relevant parameters for the best model generated by kuenm.

**Settings**	**RM**	**FC**	Trian. AUC	**avg.test.AUC**	**AICc**	**delta.AICc**
1.1_LQPT	1.1	LQPT	0.9904	0.978	1529.055	0
