# Supplementary material for: Prediction of Suitable Habitat Distribution of Cryptosphaeria pullmanensis in the World and China under Climate Change
Source: J Fungi (Basel). 2023 Jul 11;9(7):739. doi: 10.3390/jof9070739 (PMC10381404; doi:10.3390/jof9070739)
Supplement: Supplementary file 1 [file jof-09-00739-s001.zip › Table S5 Percent contribution and permutation importance of 11 variables.pdf]

Table S5. Percent contribution and permutation importance of 11 variables

Code	Percent contribution	Permutation importance
bio1	35.4	31.7
bio18	29.4	18.3
bio14	15.3	42.6
gm_lc_v3	6	0.4
elev	5.4	0.4
t-silt	3.1	3.4
t-teb	2	0.1
t-ece	1.1	0
t-grave	1	1.7
aspect	0.8	0.6
slope	0.5	0.8
