# Supplementary material for: Prediction of Suitable Habitat Distribution of Cryptosphaeria pullmanensis in the World and China under Climate Change
Source: J Fungi (Basel). 2023 Jul 11;9(7):739. doi: 10.3390/jof9070739 (PMC10381404; doi:10.3390/jof9070739)
Supplement: Supplementary file 1 [file jof-09-00739-s001.zip › Table S6 The proportion of suitable areas for growing different grades of C. pullmanensis around world.pdf]

Table S6. The proportion of suitable areas for growing different grades of *C. pullmanensis* around world

Scenario	Period	High suitable area		Medium suitable area		Low suitable area		Total suitable area	
		Area ($\times 10^6$/km^2)	Change (%)	Area ($\times 10^6$/km^2)	Change (%)	Area ($\times 10^6$/km^2)	Change (%)	Area ($\times 10^6$/km^2)	Change (%)
Current	1970-2000	0.59		1.42		3.37		5.38	
SSP126	2021-2040/2030s	0.61	3.39	1.05	-26.06	2.7	-19.88	4.36	-18.96
	2041-2060/2050s	0.64	8.47	1.16	-18.31	3.13	-7.12	4.93	-8.36
	2061-2080/2070s	0.63	6.78	1.28	-9.86	3.23	-4.15	5.14	-4.46
	2081-2100/2090s	0.61	3.39	1.12	-21.13	3.12	-7.42	4.85	-9.85
SSP370	2021-2040/2030s	0.51	-13.56	1.11	-21.83	3.03	-10.09	4.65	-13.57
	2041-2060/2050s	0.53	-10.17	1.03	-27.46	2.83	-16.02	4.39	-18.40
	2061-2080/2070s	0.48	-18.64	1.12	-21.13	3.14	-6.82	4.74	-11.90
	2081-2100/2090s	0.45	-23.73	1.04	-26.76	2.47	-26.71	3.96	-26.39
SSP585	2021-2040/2030s	0.68	15.25	1.1	-22.54	3.39	0.59	5.17	-3.90
	2041-2060/2050s	0.52	-11.86	1.09	-23.24	3.06	-9.20	4.67	-13.20
	2061-2080/2070s	0.61	3.39	0.95	-33.10	2.8	-18.63	4.36	-18.96
	2081-2100/2090s	0.29	-50.85	1.05	-26.06	1.05	-68.84	2.39	-55.58
