# Supplementary material for: Prediction of Suitable Habitat Distribution of Cryptosphaeria pullmanensis in the World and China under Climate Change
Source: J Fungi (Basel). 2023 Jul 11;9(7):739. doi: 10.3390/jof9070739 (PMC10381404; doi:10.3390/jof9070739)
Supplement: Supplementary file 1 [file jof-09-00739-s001.zip › Table S7 The proportion of suitable areas for growing different grades of C. pullmanensis in China.pdf]

Table S7. The proportion of suitable areas for growing different grades of *C. pullmanensis* in China.

Scenario	Period	High suitable area		Medium suitable area		Low suitable area		Total suitable area	
		Area (×10^4/km^2)	Change (%)	Area (×10^4/km^2)	Change (%)	Area (×10^4/km^2)	Change (%)	Area (×10^4/km^2)	Change (%)
Current	1970-2000	24.96		43.89		99.07		167.92	
SSP126	2021-2040/2030s	30	20.19	34.77	-20.78	74.47	-24.83	139.24	-17.08
	2041-2060/2050s	37.55	50.44	33.89	-22.78	109.12	10.14	180.56	7.53
	2061-2080/2070s	35.2	41.03	49.59	12.99	98.87	-0.20	183.66	9.37
	2081-2100/2090s	29.21	17.03	44.48	1.34	107.24	8.25	180.93	7.75
SSP370	2021-2040/2030s	26.69	6.93	38.69	-11.85	91.95	-7.19	157.33	-6.31
	2041-2060/2050s	32.12	28.69	39.7	-9.55	113.42	14.48	185.24	10.31
	2061-2080/2070s	28.92	15.87	53.34	21.53	121.99	23.14	204.25	21.64
	2081-2100/2090s	30.6	22.60	47.42	8.04	97.8	-1.28	175.82	4.70
SSP585	2021-2040/2030s	44.51	78.33	37.76	-13.97	120.4	21.53	202.67	20.69
	2041-2060/2050s	29.27	17.27	46.65	6.29	112.66	13.72	188.58	12.30
	2061-2080/2070s	43.41	73.92	35.55	-19.00	101.12	1.82	180.08	7.24
	2081-2100/2090s	20.55	-17.67	54.06	23.17	80.95	-18.29	155.56	-7.36
