# Supplementary material for: Prediction of Suitable Habitat Distribution of Cryptosphaeria pullmanensis in the World and China under Climate Change
Source: J Fungi (Basel). 2023 Jul 11;9(7):739. doi: 10.3390/jof9070739 (PMC10381404; doi:10.3390/jof9070739)
Supplement: Supplementary file 1 [file jof-09-00739-s001.zip › Table S8. Percentage of distrbution changes of C.pumanensis between current and future climate-change scenarios in China.pdf]

Table S8. Percentage of distribution changes of *C. pullmanensis* between current and future climate-change scenarios in China

Scenario	Period	Extended area	Shrink area	Stable area
		Area (×10^4/km^2)	Area (×10^4/km^2)	Area (×10^4/km^2)
SSP126	Current-2030s	8.72	3.49	37.94
	Current-2050s	12.97	2.47	38.96
	Current-2070s	13.92	1.56	39.88
	Current-2090s	9.27	3.14	38.29
SSP370	Current-2030s	8.69	3.91	37.52
	Current-2050s	11.64	3.52	37.92
	Current-2070s	15.2	3.87	37.56
	Current-2090s	18.39	8.56	32.88
SSP585	Current-2030s	17.48	1.19	40.24
	Current-2050s	13.72	4.48	36.96
	Current-2070s	19.7	4.81	36.63
	Current-2090s	16.52	16.52	24.92
