# Supplementary figures and images for: Prediction of Suitable Habitat Distribution of Cryptosphaeria pullmanensis in the World and China under Climate Change
Source: J Fungi (Basel). 2023 Jul 11;9(7):739. doi: 10.3390/jof9070739 (PMC10381404; doi:10.3390/jof9070739)

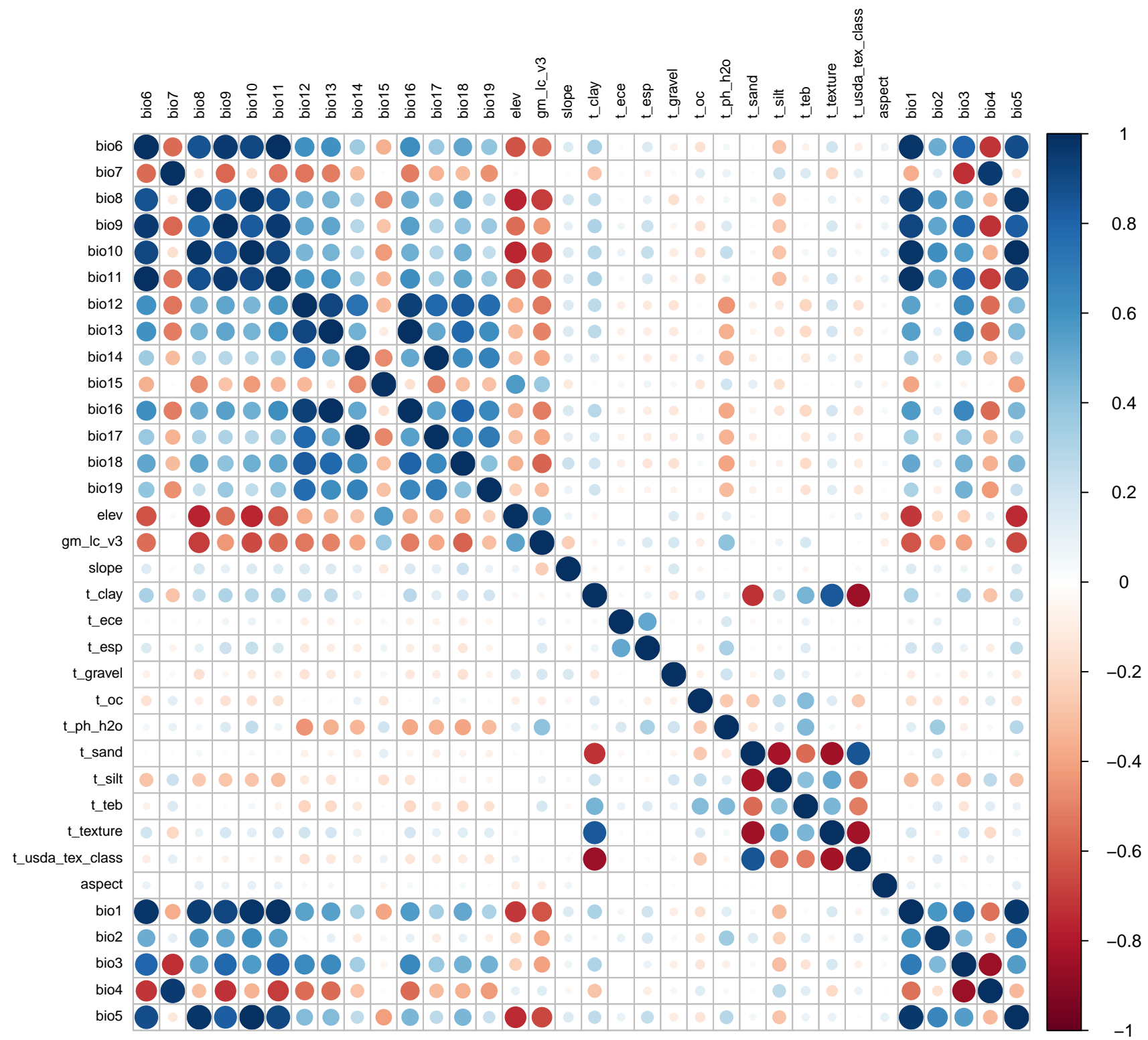

Supplement: Supplementary file 1 [file jof-09-00739-s001.zip › Figure S1 Correlation analysis of various environmental factors.pdf]

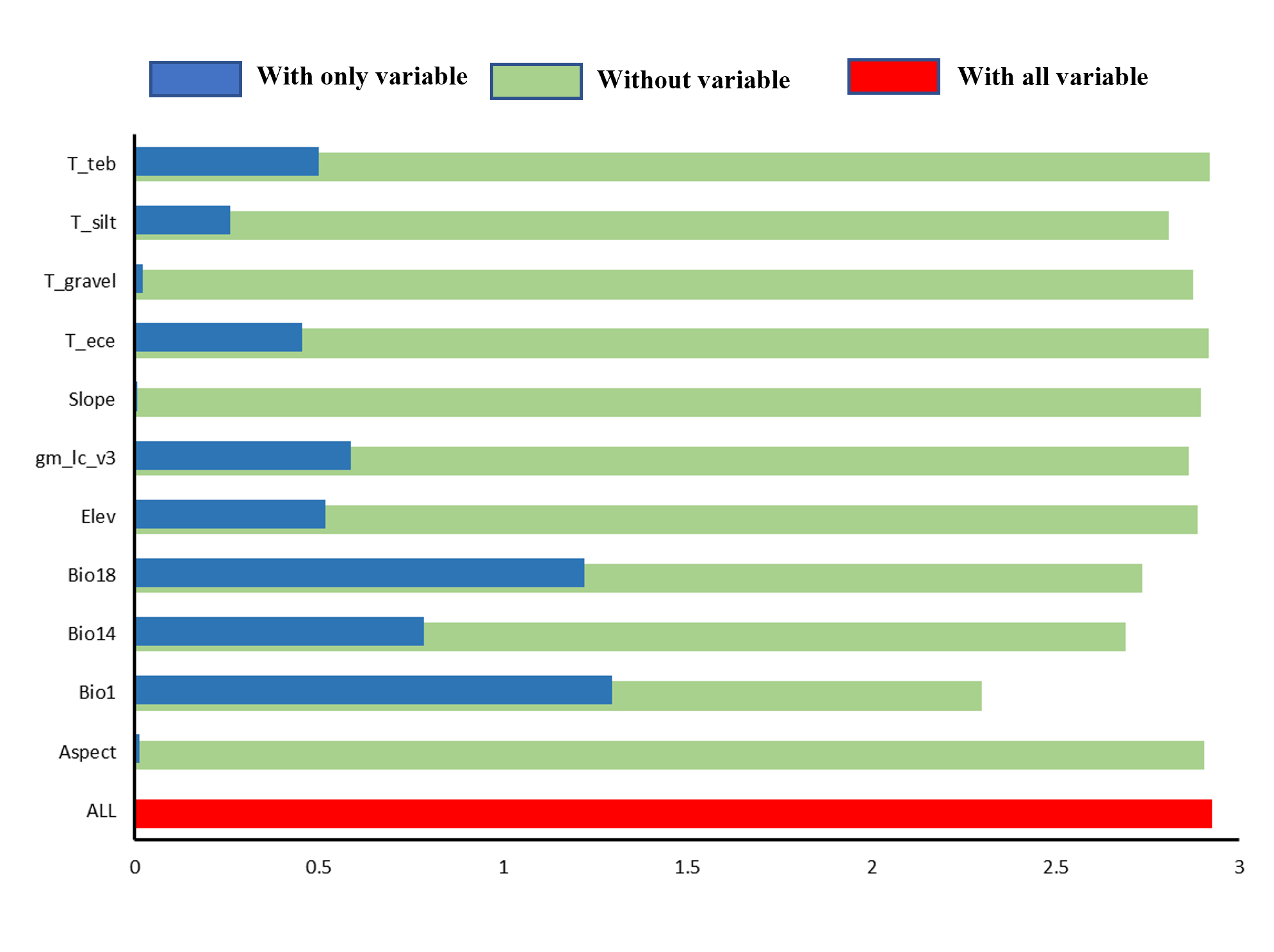

Supplement: Supplementary file 1 [file jof-09-00739-s001.zip › Figure S2. Jackknife test of variable importance Regularized training gain.tif]

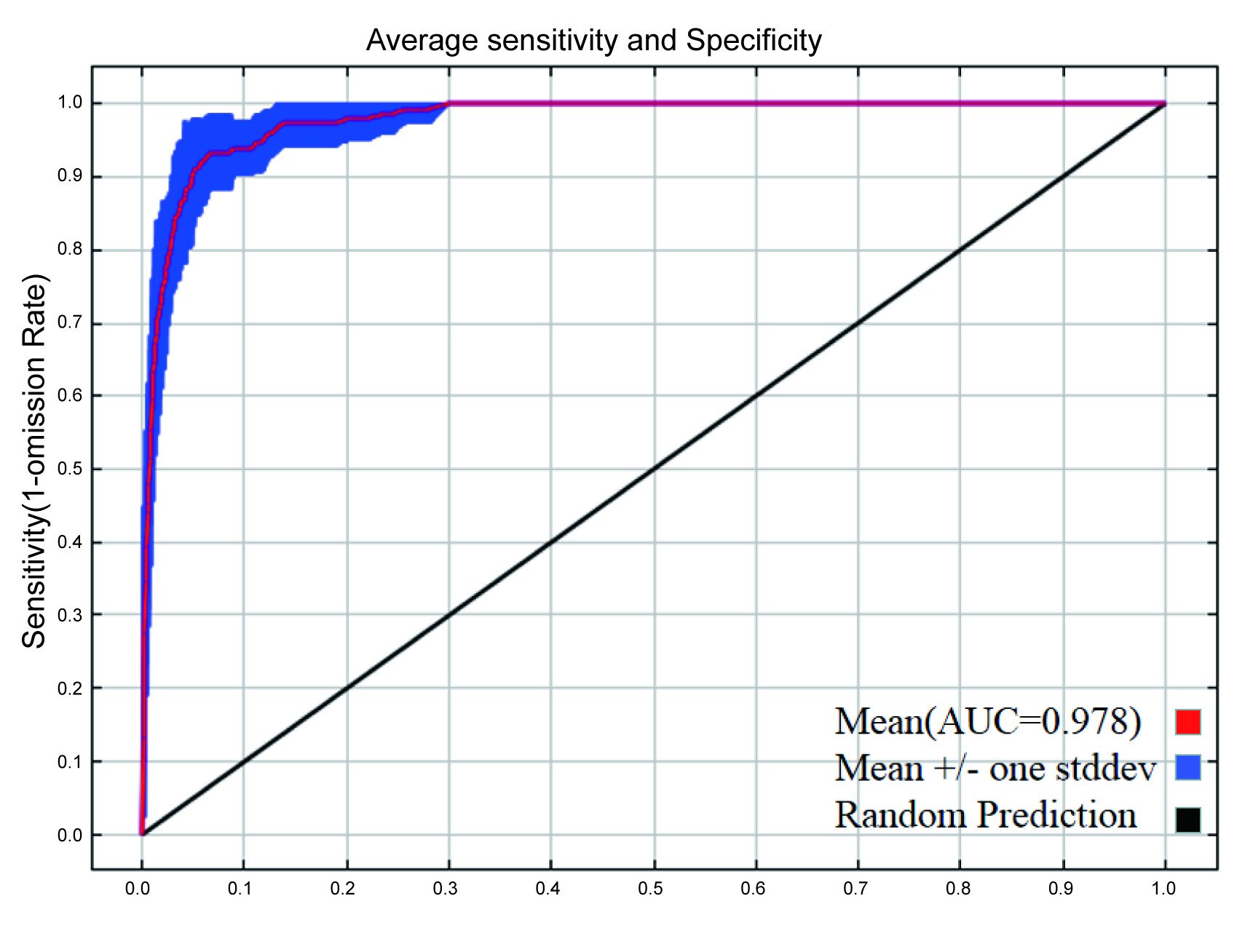

Supplement: Supplementary file 1 [file jof-09-00739-s001.zip › Figure S3 The receiver operating characteristic curve.tif]

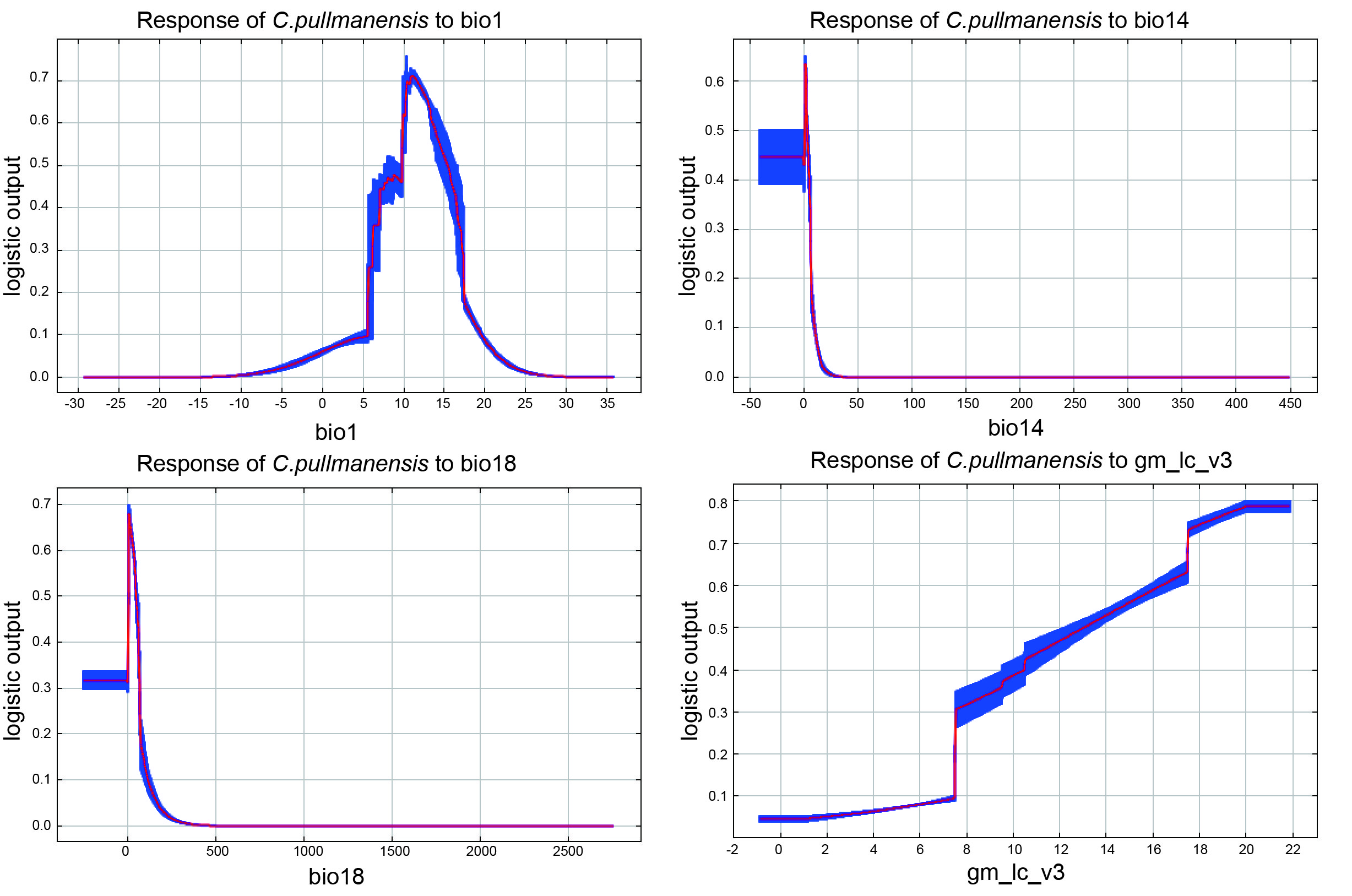

Supplement: Supplementary file 1 [file jof-09-00739-s001.zip › Figure S4 Response curves for predictiors in MaxEne model.jpg]
